# Supplementary material for: Associations of the Neutrophil-to-Lymphocyte Ratio and Platelet-to-Lymphocyte Ratio with Osteoporosis: A Meta-Analysis
Source: Diagnostics (Basel). 2022 Nov 28;12(12):2968. doi: 10.3390/diagnostics12122968 (PMC9776713; doi:10.3390/diagnostics12122968)
Supplement: Supplementary file 1 [file diagnostics-12-02968-s001.zip › diagnostics-2047821-supplementary.pdf]

Supplementary Materials

Associations of the Neutrophil-to-lymphocyte Ratio and Platelet-to-lymphocyte Ratio with Osteoporosis: A Meta-Analysis

Yu-Cheng Liu, Tzu-I Yang, Shu-Wei Huang, Yi-Jie Kuo, and Yu-Ping Chen

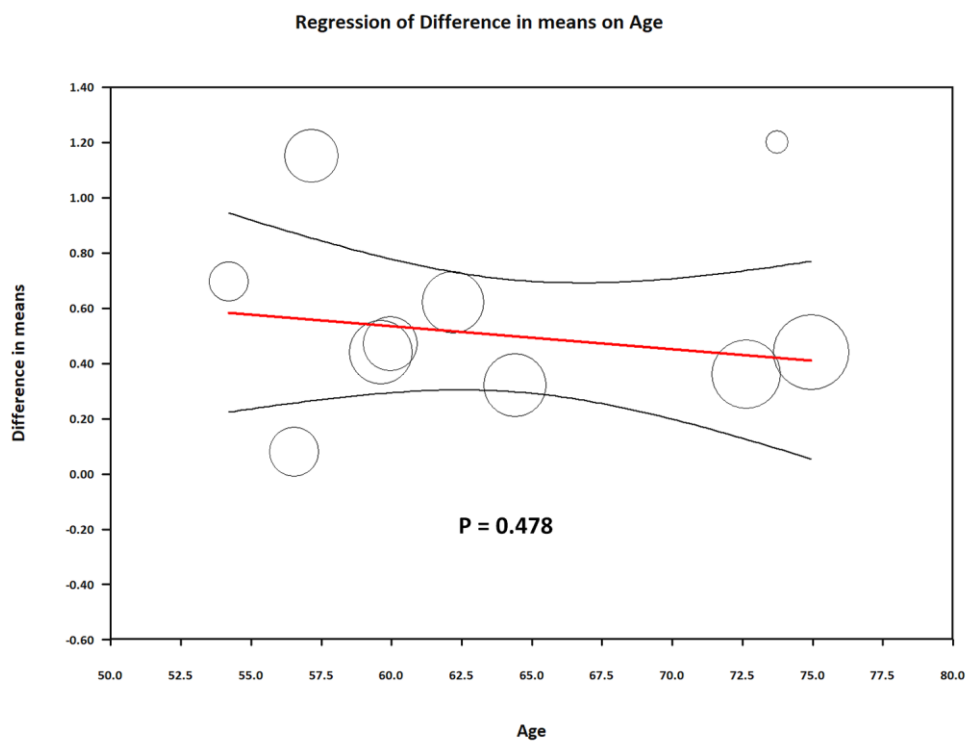

Figure S1. Meta regression of age and mean difference in NLR.

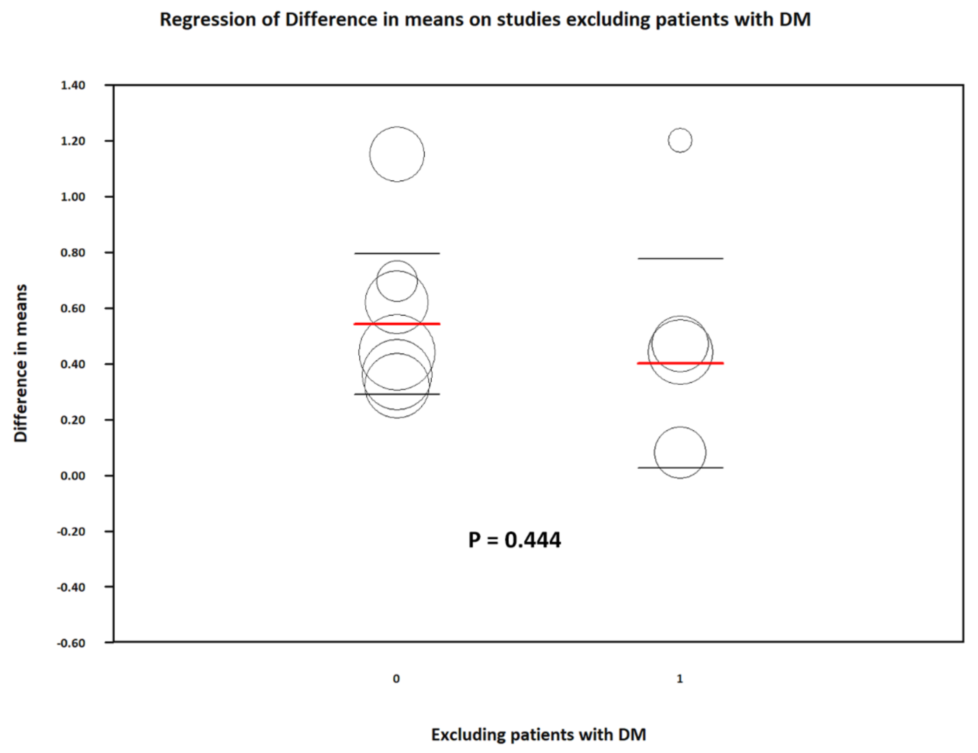

Figure S2. Meta regression of the exclusion of DM patients and mean difference in NLR. DM, diabetes mellitus; 1, DM patients excluded; 0, DM patients not excluded.

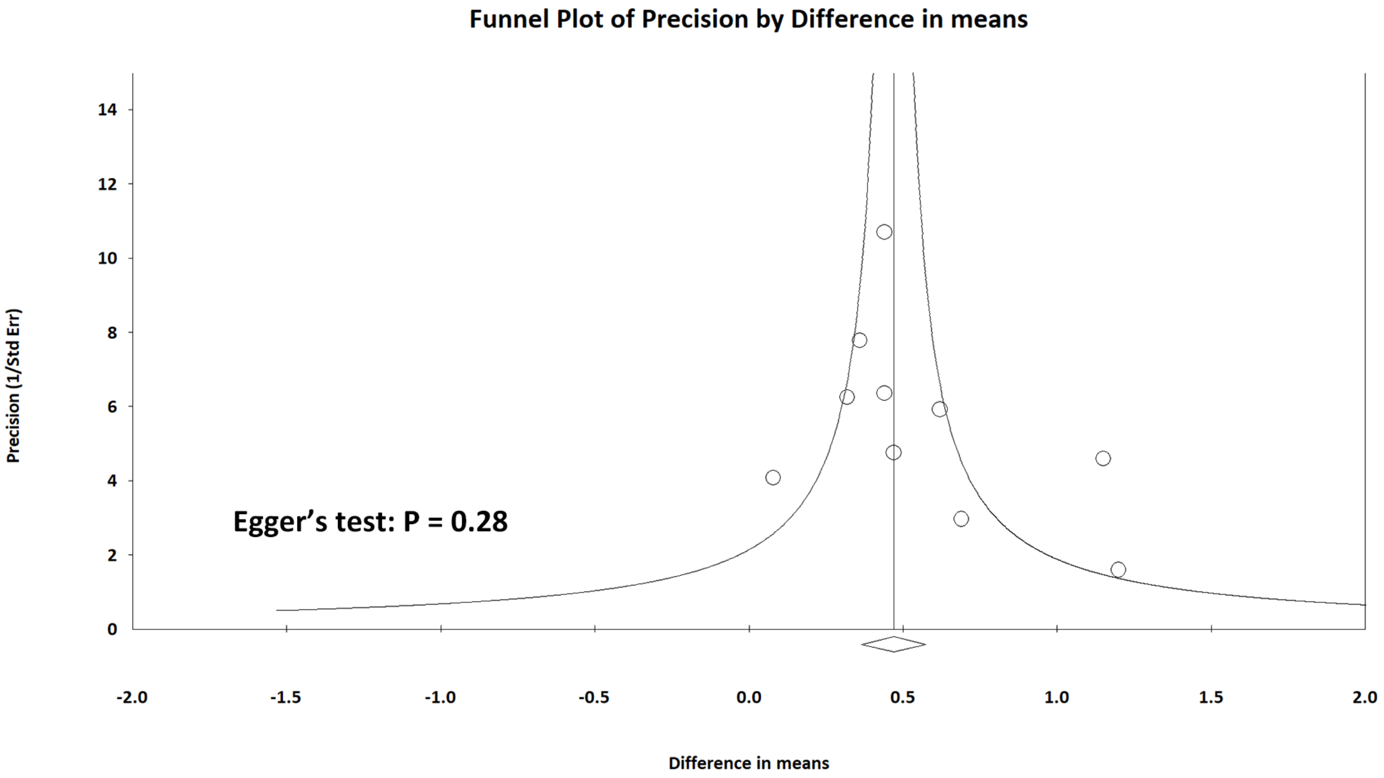

Figure S3. Funnel plot of the studies comparing NLR between the osteoporosis and normal BMD groups.

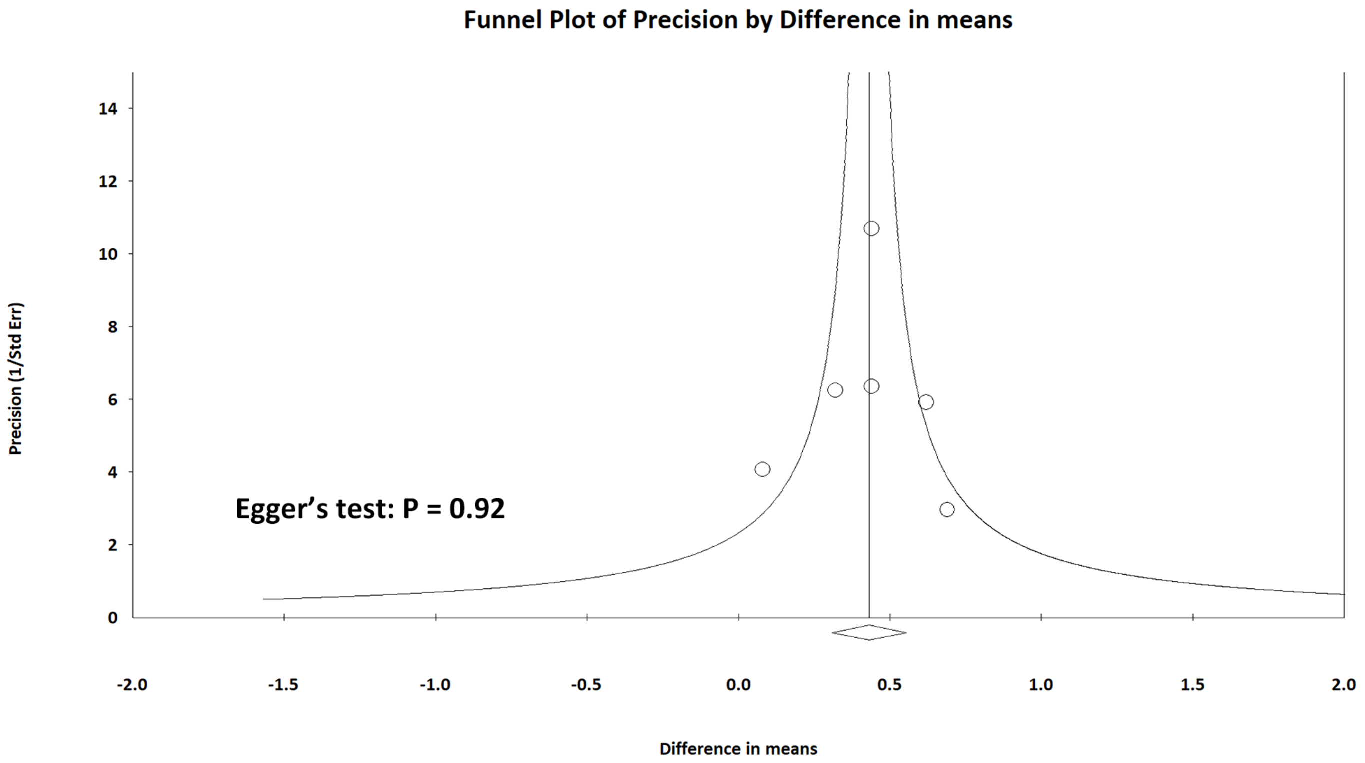

Figure S4. Funnel plot of the studies comparing NLR between the osteoporosis and normal BMD groups in postmenopausal women.

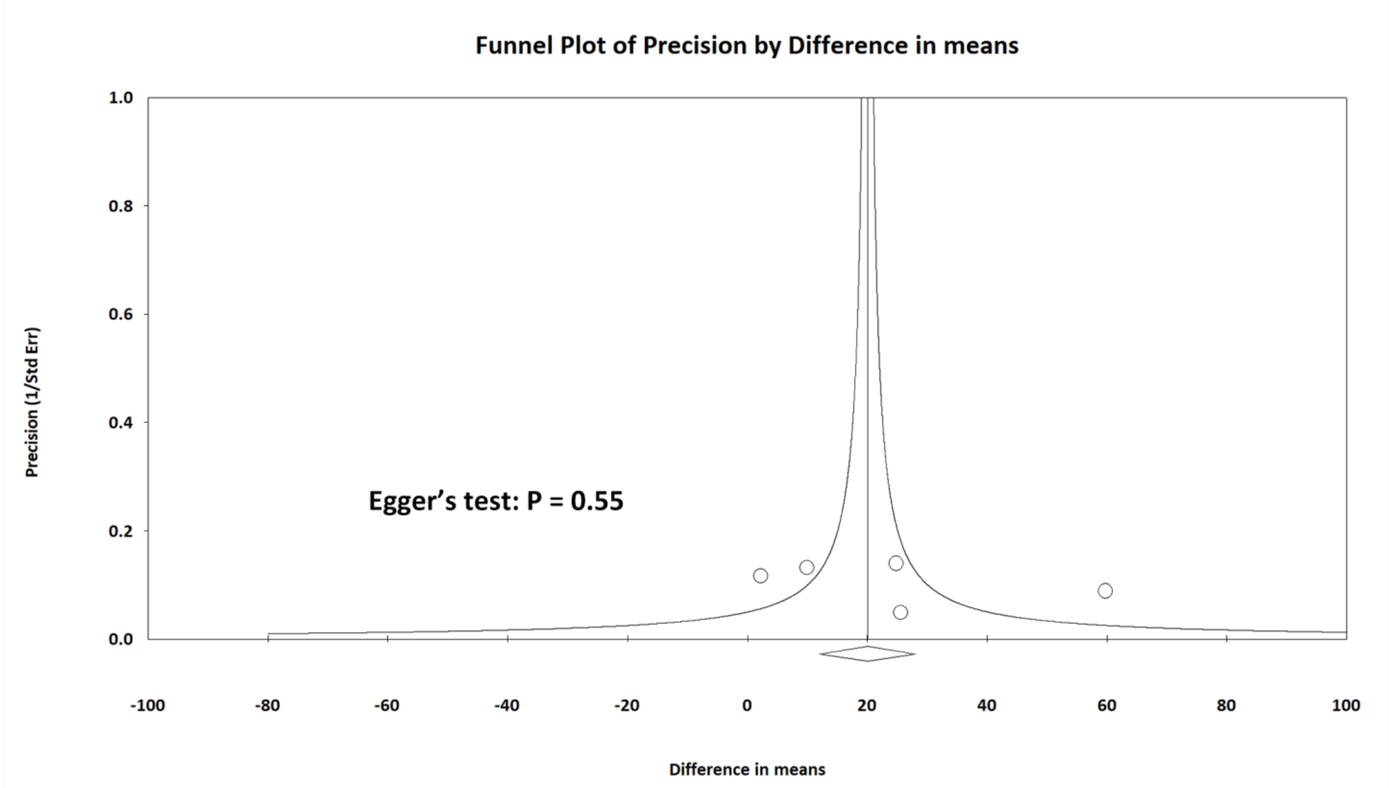

Figure S5. Funnel plot of the studies comparing PLR between the osteoporosis and normal BMD groups.

Table S1: Methodological quality of the included studies.

Author (year)	Risk of bias assessment based on the **modified Newcastle–Ottawa Scale**			
	Selection	Comparability	Outcome	Total score (quality)
Eroglu et al. (2019) [18]	***	**	***	8 (high)
Gao et al. (2019) [19]	***	**	***	8 (high)
Huang et al. (2016) [20]	***	--[1]	**	5 (moderate)
Kale et al. (2021) [21]	***	*	***	7 (high)
Onalan et al. (2020) [22]	***	*	***	7 (high)
Ozturk et al. (2013) [23]	***	*	***	7 (high)
Qin et al. (2021) [24]	***	**	***	8 (high)
Salmani et al. (2021) [17]	***	--[1]	***	6 (moderate)
Seo et al. (2009) [25]	***	*	***	7 (high)
Yu et al. (2015) [26]	***	**	***	8 (high)

[1] The original study had poor baseline control for potential confounding factors. Each * represents scoring 1 point for the according questions in each domain.
